# Assessments of productive performance, eggshell quality, excreta moisture, and incubation traits of laying breeder hens fed a proprietary blend of Quillaja and Yucca

**DOI:** 10.3389/fvets.2022.1069295

**Published:** 2023-01-18

**Authors:** Otoniel F. Souza, Carine B. Adams, Jessica C. Agilar, Valeria Biselo, Renius O. Mello, Luis G. Gomez, Sandra Bonaspetti, Catarina Stefanello

**Affiliations:** ^1^Department of Animal Science, Federal University of Santa Maria, Santa Maria, Brazil; ^2^Department of Food Technology and Science, Federal University of Santa Maria, Santa Maria, Brazil; ^3^Phibro Animal Health Corporation, Teaneck, NJ, United States; ^4^Phibro Animal Health Corporation, Campinas, Brazil

**Keywords:** cuticle quality, eggshell, hen, polyphenol, saponin

## Abstract

A study was conducted to evaluate performance, eggshell quality, nutrient metabolizability, and incubation traits of laying breeder hens fed diets supplemented with an additive containing polyphenols and saponins of a proprietary blend from *Quillaja saponaria* and *Yucca schidigera* (QY) biomass. Hens were fed 4 feeds in 5 periods of 28 days each from 30 to 49 weeks of age. Experimental feeds were a Control diet; Control + virginiamycin (33 g/ton); Control + QY (250 g/ton) and Control + virginiamycin + QY. A total of 40 White Plymouth Rock and 44 Rhode Island Red breeder hens were allocated in individual cages using a completely randomized block design with 21 replicates. Performance parameters, evaluated per period, were egg production, egg weight, FCR, egg mass, and culled eggs. All eggs were collected in the last 4 days of each period to evaluate specific egg weight, percentage of albumen, yolk and shell, and Haugh unit as well as cuticle quality, shell strength, and shell thickness. At the end of the experiment, nutrient metabolizability assessment and four incubations were conducted. There were no interactions between diet and period in all evaluated responses (*P* > 0.05). Experimental diets did not affect daily egg production, egg weight, and egg mass as well as Haugh unit, yolk and albumen percentage, and yolk color (*P* > 0.05). However, hens fed Control + QY produced eggs with better shell strength, shell thickness, and cuticle quality than hens fed the Control (*P* < 0.05). Hens fed Control + QY or Control + virginiamycin + QY had lower culled eggs, better FCR and higher egg specific weight, shell percentage, and yolk strength compared to breeder hens fed the Control (*P* < 0.05). In general, hens fed QY achieved enhanced performance and egg quality compared to virginiamycin. In conclusion, laying breeder hens fed diets supplemented with Quillaja and Yucca additive, from 30 to 49 weeks of age, maintained their productive performance, had improved eggshell and cuticle quality and reduced culled, dirty and contaminated eggs.

## 1. Introduction

The use of plant-based additives in poultry diets, as the supplementation of natural extracts, has increased in recent years due to the possibility of reducing production costs while improving productive and reproductive performance (1). Many of these additives, which have already proven effective in the nutrition and intestinal health of broilers, have then been increasingly evaluated or registered for use in diets for laying hens, broiler breeders, turkeys, and swine.

Under a scenario of high price of supplies and ingredients, along with the greater control of additives used in animal diets, natural biomasses composed by bioactive compounds, saponins, and polyphenols, have been evaluated more in recent years as additives in broiler feeds. These natural compounds have antimicrobial, immunomodulatory, and health-promoting properties (2, 3) without accumulating residues in meat or eggs, and without needing a withdrawal period. For this reason, plant-based additives have been replacing in-feed antibiotics, which have been banned in most of the companies (4).

The extract obtained from processing the bark of *Quillaja saponaria*, a native tree from Chile, presents saponins with a triterpenoid structure that showed positive results in preventing microorganism infection in the cell wall, improving membrane integrity, and presenting antibacterial activities when used in poultry feeds (5). It is also known to contain polyphenol compounds such as piscidic, vanillic, and p-coumaric acid with anti-inflammatory and antioxidant properties (6). On the other hand, Yucca extract is an additive obtained after processing *Yucca schidigera* logs, a plant native to the desert in the southwestern United States and Mexico, which has been the source of many polyphenolic compounds, such as resveratrol and yuccaols (7).

The combination of Quillaja and Yucca in an additive containing polyphenols and saponins of a proprietary blend from *Quillaja saponaria* and *Yucca schidigera* biomass has been widely used in animal diets in the United States, Asia, South America, and some other areas for over 20 years. The use of this additive in diets for broilers resulted in increased body weight gain and improved feed conversion, which was accompanied by greater ileal digestibility of dry matter, energy, and nitrogen as well as greater villus height and improved intestinal permeability (8). Additionally, the beneficial effects of Quillaja and Yucca for broilers have been previously reported to reduce the negative effects of necrotic enteritis, having antioxidant and anti-inflammatory effects (9–12), and decreasing ammonia and moisture excretion (13, 14).

The use of the Quillaja and Yucca blend for breeder or laying hens is recent, and there is no previous report in the literature evaluating the effects of polyphenols and saponins in diets for laying breeder hens. Therefore, this study was conducted to evaluate the effects of an additive containing polyphenols and saponins of a proprietary blend from *Q. saponaria* and *Y. schidigera* (QY) biomass for laying hens from 30 to 49 weeks of age. The effects of the QY additive, virginiamycin, or the combination of both were evaluated on productive performance, egg quality, and eggshell quality as well as pH and moisture of excreta, nutrient metabolizability, and incubation parameters.

## 2. Material and methods

The Ethics and Research Committee of the Federal University of Santa Maria (Santa Maria, RS, Brazil) approved all procedures used in this study.

### 2.1. Housing and experimental diets

A total of 40 White Plymouth Rock and 44 Rhode Island Red laying breeder hens were allocated at 27 weeks of age and subjected to a 4-weeks adjustment to the experimental diets (from 27 to 30 weeks). Hens were individually weighed and standardized by body weight and egg production before starting the experiment (SD of 5% of the mean weight of all birds). At 30 weeks, hens were placed in individual wire cages (0.33 m length × 0.46 m deep × 0.40 m height) and fed the experimental diets from 30 to 49 weeks of age. Feed and water were provided *ad libitum*. The lighting program was 16L:8D cycle.

Measurements were taken by periods defined as 30–33, 34–37, 38–41, 42–45, and 46–49 weeks of age. Hens were allocated in individual cages and distributed in a randomized complete block design with 10 White Plymouth Rock and 11 Rhode Island Red hens per treatment (21 hens per treatment). Laying breeder hens were fed 4 experimental diets, with a Control basal diet without additives; control supplemented with virginiamycin 16.5 ppm (33 g/ton; Stafac 500^®^ Phibro do Brasil, Campinas, SP, Brazil); control supplemented with QY product (250 g/ton) and control supplemented with 16.5 ppm virginiamycin + 250 g/ton QY product (Control + virginiamycin + QY). Experimental mash feeds were formulated with corn, soybean meal, and wheat bran (Table 1). The control diet was produced in a 400-kg horizontal mixer, then the experimental feeds supplemented with virginiamycin, QY product, virginiamycin + QY product were mixed in a Y mixer with 40 kg capacity. Celite at 1% was supplemented in each experimental feed as indigestible marker and provided 3 days before the excreta collection.

**Table 1:** Ingredient and nutrient composition of the control diets, as-is.

**Item**	**Control diet (from 30 to 49 weeks of age)**
**Ingredients, %**	
Corn	59.21
Soybean meal, 46 %CP	19.96
Wheat bran	4.00
Soybean oil	2.40
Dicalcium phosphate	1.39
Limestone	11.93
Salt	0.40
DL-Methionine, 99%	0.30
Lysine sulfate, 60%	0.17
L-Threonine, 98.5%	0.09
Mineral and Vitamin premix^1^	0.15
**Nutrient and energy composition, % or as shown**	
AME, kcal/kg	2,750
Crude protein	14.71
Calcium	4.00
Available phosphorus	0.33
Total phosphorus	0.51
Sodium	0.17
Potassium	0.60
Chloride	0.31
Dig. Lys^2^	0.74
Dig. Met + Cys	0.70
Dig. Thr	0.58
Dig. Trp	0.16
Dig. Arg	0.85
Dig. Val	0.58
Dig. Ile	0.53
Dig. Leu	1.18

1 Composition per kilogram of feed: vitamin A, 8,000 UI; vitamin D_3_, 2,000 UI; vitamin E, 30 UI; vitamin K_3_, 2 mg; thiamine, 2 mg; riboflavin, 6 mg; pyridoxine, 2.5 mg; cyanocobalamin, 0.012 mg, pantothenic acid, 15 mg; niacin, 35 mg; folic acid, 1 mg; biotin, 0.08 mg; iron, 40 mg; zinc, 80 mg; manganese, 80 mg; copper, 10 mg; iodine, 0.7 mg; selenium, 0.3 mg.

2 Ratios of digestible amino acids to digestible Lys will be maintained at TSAA 0.94; Thr 0.77; Val 0.77; Trp 0.20; Arg 1.13; Ile 0.67.

As described by Bafundo et al. (10), QY product (Magni-Phi, Phibro Animal Health Corp., Teaneck, NJ, US) consists of 100% ground plant material derived from *Q. saponaria* trees and *Y. schidigera* plants formulated in a proprietary ratio, where Quillaja is the major component. The product does not contain excipients or carriers; thus, active ingredients of the product comprise unextracted and unaltered Quillaja saponins supplemented with the saponins naturally contained in Yucca. These substances constitute 4% of the total product.

### 2.2. Hen performance and egg quality

Daily egg production (%), feed intake (g/bird/day), feed conversion (FCR, kg feed/dozen eggs and kg feed/kg eggs), egg mass, and culled eggs (%) were recorded in 5 periods of 28 days. The culled eggs (%) were considered as eggs that are porous, cracked, broken, or thin shell that are not suitable for incubation. Dirty eggs (%) were also daily evaluated per cage and grouped by period.

At the end of each 28-day period, average egg weight, albumen height, specific egg weight, shell percentage, and shell thickness were assessed in 4 consecutive days. Albumen and yolk weights were recorded to determine their percentage to the entire egg. Yolk quality was determined by measuring yolk height (YH) and yolk width (YW), thus yolk index (YI) was calculated as the ratio between YH and YW.

The yolk color measurements were conducted using a colorimeter (Konica Minolta CM-700D, Japan) to objectively measure L^*^ (lightness), a^*^ (redness), and b^*^ (yellowness). The apparatus was standardized against a white tile before each measurement. Color values of yolk were measured in all eggs per experimental unit (2 times per egg) during the egg quality evaluation in each 28-day period.

All intact eggs from each experimental unit were identified and weighed individually in a precision scale (0.001 g; Bioscale, São Paulo, Brazil). The specific weight test was conducted using the flotation method with seven saline solutions measured with densimeter oil, ranging from 1.070 to 1.094 g/cm^3^ with a variation of 0.004 g/cm^3^ for each solution. After the test, all eggs were used to determine albumen height and percentages of albumen, yolk, and shell. The measurement in millimeters (mm) was related to egg weight to determine the Haugh unit: HU log 100(H + 7.57–1.7 W^0.37^), in which H = albumen height (mm) and W = egg weight (g).

Shells were washed and dried at room temperature for 72 h, and weighed using a precision digital scale (0.001 g; Bioscale, São Paulo, Brazil). After weighing the shells, shell thickness was measured at 3 points in the central region of each shell using a manual micrometer (Mitutoyo Sul Americana, São Paulo, Brazil). All intact eggs produced at the end of each cycle were used for the measurement of shell strength (kgf), which was obtained with a texturometer TA.XT2 Texture Analyzer with Cyln Stainless 6-mm probe (Texture Technologies Corp. and Stable Micro Systems Ltd., Hamilton, MA, US) according to Stefanello et al. (15).

After shell strength evaluation, the egg was broken to measure the yolk vitelline membrane strength (kgf), also conducted with a texturometer TA.XT2 Texture Analyzer with Cyln Stainless 2-mm probe. The yolk vitelline membrane strength followed the method described by Souza et al. (16).

### 2.3. Cuticle quality

The cuticle coverage was assessed for all eggs before shell strength measurement at the end of each 28-day period. Staining was achieved by immersion of unwashed eggs in an aqueous solution containing Tartrazine and Green S (Metaquímica, Jaraguá do Sul, SC, Brazil). Then, the shell was rinsed in water to remove excess dye prior to drying (17, 18). Quantification was done with a colorimeter (colorimeter Konica Minolta CM-700D, Japan) by measuring the color difference (L^*^a^*^b^*^) of the eggshell at four points in the central region of each egg before and after staining of the cuticle (defined as Δ). Color difference or cuticle quality was then expressed as a single numerical value (ΔE${}_{\text{ab}}^{*}$). A higher cuticle quality denotes a higher staining affinity and hence more cuticle coverage. The ΔE${}_{\text{ab}}^{*}$ is defined by the following formula:


$$\Delta E^{*}{}_{ab}=\sqrt{\left[{\left(\Delta L^{*}\right)}^{2}+{\left(\Delta a^{*}\right)}^{2}+\;{\left(\Delta b^{*}\right)}^{2}\right]}$$

### 2.4. Excreta pH, moisture, and total tract metabolizability

At the end of each 28-day cycle, excreta were collected in trays covered with plastic that were placed under the cages, positioned individually in each experimental unit. To obtain excreta pH, fresh 10 g of excreta was homogenized with 100 mL distilled water for 30 min as described by Brauer-Vigoderis et al. (19). Then, the pH was measured (twice per sample) with a pH meter equipped with an electrode (InLab 413 SG; Mettler-Toledo GmbH).

Excreta samples from each experimental unit were collected, mixed, pooled by cage and stored at −20°C until analysis. Subsequently, excreta were weighed and dried in a forced air oven at 55°C (Fisher Isotemp Oven, Waltham, MA, US), ground to pass a 0.5 mm screen (Tecnal, R-TE-648, São Paulo, SP, Brazil), and analyzed. Moisture analysis of excreta was performed after oven drying the samples at 105°C for 16 h (method 934.01) (20). Gross energy was determined using an adiabatic bomb calorimeter (Parr Instrument Company, 6,400 Calorimeter, Moline, IL, US) and nitrogen used a combustion nitrogen analyzer *via* dry combustion method (Thermo-Finnigan Flash EA 1112, Waltham, MA, US). Acid insoluble ash in the diets and excreta samples was determined as described by Souza et al. (16). Apparent metabolizable energy (AME, in kcal/kg, on DM basis) and the coefficient of apparent total tract metabolizability of dry matter, nitrogen, and energy (in %, on DM basis) were calculated as previous reported by Kong and Adeola (21) and Souza et al. (16).

### 2.5. Incubation variables

Eggs were incubated for 4 consecutive weeks at the end of the experiment, using the same incubator and hatching equipment to evaluate hatching capacity from fertile eggs, embryonic mortality, and pipped eggs. At 49 weeks, semen was collected from breeder males (White Plymouth Rock or Rhode Island Red) that were kept separate from the females in individual cages. Semen was pooled per breed (*n* = 10 per breed) and diluted in a 2:1 ratio of physiological solution immediately before the insemination, once a week.

All eggs were collected daily, classified, separated for incubation for each experimental unit, and stored for 7 days in a controlled temperature room [18–20°C and 75–80% RH (relative humidity)]. Incubation was conducted in a commercial multi-stage incubator (capacity = 5,760 eggs; Casp, Amparo, São Paulo, SP, Brazil) at 37.5°C and 60% RH. At 18 days, eggs were transferred to the hatching equipment (capacity = 4,320 eggs) which was calibrated at 36.5°C and 65% RH.

Hatchability of fertile eggs (%) was expressed as the number of hatching chicks per fertile eggs set. Embryodiagnosis was conducted in eggs from which hatching did not occur to evaluate the hatching rate from fertile eggs and embryonic mortality. In this evaluation, contaminated eggs (%) were the number of contaminated eggs per total egg set. Embryo mortality was calculated as a percentage of fertile eggs and estimated at 1st, 2nd, and 3rd week of incubation, being classified as early, middle, and late dead, respectively. Pipped eggs were embryos still alive that broke the shell, but did not emerge by the time of the chick's removal from the hatching equipment.

### 2.6. Statistical analysis

Data were analyzed using a normality test and transformed using the arcsine square root percentage [z = arcsin (sqrt (y/100))] whenever it was not normally distributed. Data were subjected to analysis of variance using the MIXED procedure of SAS (22) with each one of the 28-day periods as repeated measures. The effect of diet was fixed, the effect of periods was repeated, and the interaction between diet vs. period was also conducted. Means were compared by the Student-Newman-Keuls Test (*P* < 0.05). The covariance structures of mixed linear model were chosen based on the Akaike criteria.

## 3. Results

There were no interactions between dietary feed additives and period for all obtained responses. Therefore, data are presented as main factors throughout this study. Productive performance of laying breeder hens fed diets supplemented with virginiamycin, Quillaja and Yucca additive, or the combination of both in the 30 to 49-week-old egg-laying period is presented in Table 2. Almost all of the evaluated parameters were affected by the period, divided into 30–33, 34–37, 38–41, 42–45, and 46–49 weeks of age (*P* < 0.05); however, the percentage of dirty eggs and shell percentage were not affected by the hens' age. Egg production, egg mass, and FCR decreased as hens aged (*P* < 0.05), while the egg weight increased as hens aged. Hens fed Control + QY or Control + virginiamycin + QY had lower culled and dirty eggs compared to breeder hens fed the Control (*P* < 0.05).

**Table 2:** Productive performance of laying breeder hens fed diets supplemented with an additive from *Quillaja saponaria* and *Yucca schidigera* (QY) from 30 and 49 weeks of age.

**Item**	**EP^1^, %**	**FI^2^, g/hen/d**	**Culled eggs, %**	**Dirty eggs, %**	**Egg mass, g**	**FCR^3^, kg/kg**	**FCR, kg/dz**	**Egg weight, g**
**Diets**								
Control	80.2	121^a^	18.0^a^	5.7^a^	44.0	2.80^a^	1.84^a^	54.9
Control + virginiamycin^4^	81.2	121^a^	16.5^a^	4.2^ab^	44.6	2.76^ab^	1.81^ab^	55.0
Control + QY	81.9	117^b^	7.2^b^	2.5^b^	45.2	2.62^b^	1.74^b^	55.3
Control + virginiamycin + QY	83.2	119^ab^	7.5^b^	3.5^ab^	45.9	2.63^b^	1.74^b^	55.2
**Periods, weeks**								
30–33	87.9^a^	124^a^	8.7^b^	4.4	46.6^a^	2.70^ab^	1.71^b^	52.8^c^
34–37	83.5^b^	123^a^	11.2^ab^	5.2	45.4^ab^	2.74^a^	1.78^b^	54.4^b^
38–41	82.2^b^	121^a^	13.5^a^	3.6	45.3^ab^	2.74^a^	1.81^ab^	55.1^b^
42–45	77.7^c^	122^a^	13.7^a^	3.7	43.8^b^	2.81^a^	1.91^a^	56.4^a^
46–49	76.7^c^	108^b^	14.3^a^	3.1	43.6^b^	2.52^b^	1.71^b^	56.8^a^
SEM	0.48	0.54	0.72	0.32	0.29	0.023	0.015	0.19
***P*** **-value**								
Diet	0.115	0.025	0.001	0.005	0.111	0.007	0.013	0.878
Period	0.001	0.001	0.051	0.289	0.289	0.001	0.001	0.001
Diet vs. period	0.993	0.363	0.998	0.922	0.922	0.934	0.921	0.996

a−c Means with different superscript letters differ (*P* ≤ 0.05) based on SNK test.

1 EP = egg production.

2 FI = feed intake.

3 FCR = feed conversion ratio. FCR (kg/kg) = kg of feed/kg eggs; FCR (kg/dz) = kg of feed/dozen eggs.

4 Virginiamycin (33 g/ton; Stafac 500^®^ Phibro do Brasil, Campinas, SP, Brazil).

As shown in Table 2, experimental feeds did not affect daily egg production, egg mass, egg weight, and Haugh unit (*P* > 0.05). However, hens fed Control + QY had lower feed intake and better FCR than hens fed the Control (*P* < 0.05). The specific egg weight, Haugh unit, albumen percentage, yolk color, shell strength, and shell thickness decreased as hens aged (*P* < 0.05) as presented in Table 3. On the other hand, culled eggs, yolk percentage, yolk index, and cuticle quality increased as hens aged (*P* < 0.05). Considering the dietary feeds, hens fed Control + QY produced eggs with better shell thickness, shell strength, and cuticle quality than hens fed the Control (*P* < 0.05). Hens fed Control + QY or Control + virginiamycin + QY had higher specific egg weight, shell percentage, and yolk index compared to hens fed the Control (*P* < 0.05) in the 30–49-week-old egg-laying period. Virginiamycin resulted in similar results of specific egg weight, shell thickness, yolk strength, and cuticle quality compared to QY supplemented feeds and the non-supplemented feed.

**Table 3:** Quality of egg, eggshell, and cuticle (ΔE^*^_ab_) of laying breeders fed diets supplemented with an additive from *Quillaja saponaria* and *Yucca schidigera* (QY) from 30 and 49 weeks of age.

**Item**	**Specific weight, g/cm^3^**	**Haugh unit**	**Albumen, %**	**Shell, %**	**Thickness, mm**	**Shell strength, kgf**	**Yolk, %**	**Yolk strength, kgf**	**Yolk index**	**Egg yolk color**			**Cuticle quality**
										**L** ^*^	**a** ^*^	**b** ^*^	ΔE*_ab_
**Diets**													
Control	1,080^b^	84.8	61.9	8.2^b^	0.33^b^	27.3^c^	29.7	7.6^b^	0.44	48.2	5.5	38.7	29.8^b^
Control + virginiamycin^1^	1,081^ab^	85.3	62.5	8.6^a^	0.35^ab^	29.1^ab^	29.7	7.7^ab^	0.44	47.8	5.7	38.8	30.9^ab^
Control + QY	1,082^a^	85.8	62.9	8.8^a^	0.36^a^	29.7^a^	30.0	7.9^a^	0.45	48.4	5.7	39.0	32.0^a^
Control + virginiamycin + QY	1,082^a^	85.9	62.8	8.6^a^	0.35^ab^	29.4^ab^	30.1	7.9^a^	0.45	47.9	5.7	38.7	31.3^ab^
**Periods, weeks**													
30 to 33	1,084^a^	87.7^a^	64.2^a^	8.7	0.37^a^	30.2^a^	28.7^c^	8.0	0.46^a^	46.4^c^	6.6^a^	38.9	27.7^d^
34 to 37	1,082^b^	89.7^a^	63.1^ab^	8.7	0.35^b^	29.4^a^	29.3^bc^	7.7	0.45^ab^	47.8^b^	6.3^ab^	40.9	29.4^cd^
38 to 41	1,081^b^	85.1^b^	61.6^b^	8.5	0.34^b^	29.2^ab^	30.2^ab^	7.7	0.45^ab^	47.7^b^	6.0^b^	39.1	31.1^bc^
42 to 45	1,080^b^	84.0^b^	62.2^b^	8.5	0.34^b^	28.7^b^	30.4^a^	7.4	0.44^b^	48.1^b^	5.5^c^	40.3	32.9^ab^
46 to 49	1,080^b^	80.5^c^	61.6^b^	8.5	0.33^c^	26.8^c^	30.8^a^	8.0	0.42^c^	50.3^a^	3.8^d^	38.8	34.0^a^
SEM	0.21	0.30	0.18	0.03	0.001	0.30	0.12	0.04	0.001	0.14	0.07	0.22	0.32
***P*** **-value**													
Diet	0.004	0.423	0.185	0.001	0.007	0.016	0.547	0.048	0.008	0.291	0.258	0.919	0.041
Period	0.001	0.001	0.001	0.144	0.001	0.006	0.001	0.070	0.001	0.001	0.001	0.101	0.001
Diet vs. period	0.558	0.999	0.994	0.948	0.999	0.999	0.999	0.802	0.931	0.762	0.994	0.172	0.999

a−d Means with different superscript letters differ (*P* ≤ 0.05) based on SNK test.

1 Virginiamycin (33 g/ton; Stafac 500^^®^^; Phibro do Brasil, Campinas, SP, Brazil).

Excreta moisture and pH are presented in Table 4. Results demonstrated that the pH of excreta samples was not influenced by the diet or age periods (*P* > 0.05). However, excreta moisture decreased when hens were fed Control + QY or Control + virginiamycin + QY compared to hens fed the Control (*P* < 0.05). Excreta moisture was not modified as hens aged (*P* > 0.05). Dietary treatments did not affect AME and total tract metabolizability of DM, nitrogen, and energy determined at 49 weeks of age (*P* > 0.05) (Table 5).

**Table 4:** Moisture and pH of excreta from laying breeder hens fed diets supplemented with an additive from *Quillaja saponaria* and *Yucca schidigera* (QY) from 30 to 49 weeks of age.

**Item**	**Excreta moisture, %**	**Excreta pH**
**Diets**		
Control	80.8^a^	7.2
Control + virginiamycin^1^	80.0^ab^	7.3
Control + QY	79.6^b^	7.4
Control + virginiamycin + QY	79.3^b^	7.3
**Periods, weeks**		
30 to 33	80.0	7.4
34 to 37	80.5	7.3
38 to 41	80.2	7.2
42 to 45	79.3	7.2
46 to 49	79.8	7.4
SEM	0.16	0.04
***P*** **-value**		
Diet	0.010	0.098
Period	0.103	0.085
Diet vs. period	0.897	0.585

a−b Means with different superscript letters differ (*P* ≤ 0.05) based on SNK test.

1 Virginiamycin (33 g/ton; Stafac 500^®^ Phibro do Brasil, Campinas, SP, Brazil).

**Table 5:** Apparent metabolizable energy and coefficients of total tract metabolizability of laying breeder hens fed diets supplemented with an additive from *Quillaja saponaria* and *Yucca schidigera* (QY) at 49 weeks of age.

**Item**	**Total tract metabolizability**			
	**AME** ^1^ **, kcal/kg**	**Dry matter**	**Nitrogen**	**Energy**
Control	3,112	0.67	0.50	0.77
Control + virginiamycin^2^	3,106	0.68	0.49	0.78
Control + QY	3,151	0.69	0.50	0.79
Control + virginiamycin + QY	3,121	0.70	0.51	0.79
SEM	17.63	0.005	0.007	0.004
*P*-value	0.815	0.348	0.786	0.771

1 AME = apparent metabolizable energy.

2 Virginiamycin (33 g/ton; Stafac 500^®^ Phibro do Brasil, Campinas, SP, Brazil).

The incubation yield conducted in four consecutive weeks after 49 weeks of age is presented in Table 6. It was observed lower contaminated and pipped eggs as well as lower early and middle mortality when hens were fed the Control + QY compared to the Control. The hatchability was not affected by virginiamycin, QY product, or the combination of both (*P* > 0.05).

**Table 6:** Incubation parameters (%) of laying breeders hens fed diets supplemented with an additive from *Quillaja saponaria* and *Yucca schidigera* (QY)^1^.

**Item**	**Hatchability^2^**	**Contaminated eggs^3^**	**Embryo mortality^4^**			
			**Early dead**	**Middle dead**	**Late dead**	**Pips**
Control	79.9	1.7^a^	1.1^ab^	1.5^a^	3.1	5.4^a^
Control + virginiamycin^5^	80.1	1.7^a^	2.1^a^	1.9^a^	4.1	3.9^ab^
Control + QY	82.2	0.0^b^	0.6^b^	0.0^b^	3.8	3.4^b^
Control + virginiamycin + QY	81.2	0.2^b^	0.6^b^	0.8^ab^	3.8	3.2^b^
SEM	0.61	0.26	0.20	0.25	0.36	0.31
*P-*value	0.514	0.026	0.023	0.038	0.795	0.052

a−b Means with different superscript letters differ (*P* ≤ 0.05) based on SNK test.

1 Eggs were incubated for 4 consecutive weeks at the end of the experiment (at 49 weeks of age) after being stored for 7 days.

2 Hatchability as a proportion total of fertile eggs, % = (number of chicks hatched/number of fertile eggs set) × 100.

3 Contaminated eggs as a percentage of total eggs set, % = (number of contaminated eggs/numbers of total egg set) × 100.

4 Early, middle, and late dead were estimated at first, second, and third week of incubation, respectively, and all data were calculated as a percentage of fertile eggs.

5 Virginiamycin (33 g/ton; Stafac 500^®^ Phibro do Brasil, Campinas, SP, Brazil).

## 4. Discussion

Virginiamycin is effective against Gram positive microorganisms that have no beneficial effects to the intestinal tract. It has been used as a growth promoter in animal feeds and in topical preparations for human and veterinary medicine. Additionally, orally administered virginiamycin acts only in the intestine, where it seems to help digestive functions, improving the intestine passage rate. No residues of virginiamycin have been reported in kidneys, livers, or muscles of chickens (23), and for this reason, the manufacturer indicates that virginiamycin does not require a withdrawal period. The use of virginiamycin is not recommended for laying hens when producing eggs for human consumption in some countries. No previous data were found in the literature evaluating virginiamycin for breeder hens. In the current study, most of the results indicated that laying breeder hens fed QY achieved enhanced performance and egg quality compared to virginiamycin, then the discussion focus on Quillaja and Yucca effects.

In-feed antibiotics are commonly used to control necrotic enteritis in broiler chickens and turkeys in many countries (24). The current trend in banning the in-feed antibiotics has gradually spread across other countries after starting in the European Union. Now, plant-based additives with immune, antimicrobial, and antioxidant functions have been used in animal feeds. Saponins from Quillaja and Yucca have been currently applied worldwide as adjuvants in vaccines, foaming agents in beverages, and used to prepare emulsions for cosmetics, detergents, and shampoos since these components have surfactant properties (2). Furthermore, QY blends have been used as dietary additives primarily for ammonia and odor control for livestock and companion animals. Recently, QY in-feed additives have been used to improve gut health, resulting in improved poultry performance mainly in the presence of intestinal challenges due to their antioxidant, anti-inflammatory, antifungal, immunostimulatory, and antibacterial properties (10, 25).

To the best of our knowledge, no publications were found evaluating QY products for laying breeder hens, and few studies previously evaluated Yucca additives for laying hens (7, 26–28), but none of these studies evaluated the QY blend. In the present study, virginiamycin and QY (250 g/ton) did not affect egg production, egg mass, and egg weight; however, the additive improved FCR. Shell thickness and shell strength were improved when hens were fed Control + QY from 30 to 49 weeks of age. Hens fed Control + QY or Control + virginiamycin + QY had improved specific egg weight, shell percentage, and yolk strength. Conversely, Alagawany et al. (7) tested an additive from Yucca (0, 100 or 150 g/ton) for Hisex Brown laying hens from 36 to 52 weeks of age and no significant differences were observed on FCR, egg weight, and eggshell quality among the tested levels. The authors observed that supplemental Yucca up to 100 or 150 g/ton diet led to improvements in egg production and egg mass as well as in immunity functions and activity of antioxidant enzymes.

In the current study, excreta moisture decreased when hens were fed QY; however, the excreta pH was not affected by the additive. Evaluating an additive from *Y. schidigera* (0–200 g/ton) supplemented in feeds for W36 laying hens from 25 to 37 weeks of age, Chepete et al. (28) did not show differences on N content, moisture, and pH of manure. Litter or manure N content, moisture, pH, and temperature are factors that contribute to ammonia formation, and about 50% of the N content of freshly excreted manure is in the uric acid form, which can be hydrolyzed, mineralized, and volatilized and very quickly transformed into ammonia (29). Evaluating a liquid *Y. schidigera* extract, Ayoub et al. (14) indicated that the additive decreased N excretion, thus improving litter quality and broiler welfare. Yucca extract was already attributed to affect N metabolism, resulting in reductions in serum urea and ammonia (7). It was suggested that non-butanol extractable Yucca components could lower urea and ammonia concentrations due to the increased rate of urea clearance as a result of altered kidney function. The Yucca extract contains a glycocomponent molecule that binds ammonia, and another active compound, which is a steroidal saponin molecule (30).

Saponins differ in the charged groups (carboxyl groups) in the side chains, and in the number of side chains (Yucca is monodesmosidal and Quillaja saponins are bidesmosidal). The high capacity for solubilizing monoglycerides, for stabilizing the oil/water interface, and for forming micelles with bile acids presented by saponins (31) indicate that saponin could improve fat emulsification and digestion. Nonetheless, percentages and composition of polyphenols in the QY blend still remain uncertain. It has been recognized that QY contains other physiologically active constituents as resveratrol, yuccaols, piscidic, vanillic, and p-coumaric acid. Oleszek et al. (30) reported 21.7 and 72.6 mg/g resveratrol content in Yucca bark and whole plant powder, respectively; while 3.2 and 10.0 mg/g yuccaols were determined in Yucca bark and whole plant powder, respectively. Maier et al. (6) identified that piscidic acid represented the major phenolic compound (75–87% of all phenolic compounds) in Quillaja extracts, ranging from 22.09 ± 0.12 to 33.99 ± 0.15 mg/g of DM. The authors also identified vanillic, p-coumaric acid, and their derivatives. Additionally, it is necessary to improve the knowledge on the effects of polyphenols from QY biomass on egg production, performance, and egg quality.

Improvements on nutrient digestibility in challenged or non-challenged broilers were previously reported when QY biomass was used (8, 10, 12). Bafundo et al. (12) observed improvements on ileal digestibility of DM, N, ash, and energy of 25-day-old broiler chickens fed 250 or 500 g/ton QY product. Stefanello et al. (8) also reported that DM, N, and energy digestibility increased in 42-day-old broilers fed 250 g/ton QY when compared to the non-supplemented diet. However, no effects of the QY additive were observed on AME and total tract metabolizability of DM, energy, and N of laying breeder hens in the current study.

The main cause of eggshell contamination is the contact of eggs with dirty surfaces immediately after eggs are laid. The measurement of cuticle quality or cuticle deposition have been used to evaluate contamination in eggs, specially from hens undergoing intestinal challenges (17). In the present study, higher cuticle deposition was found on eggs from hens fed diets supplemented with QY. Increased cuticle deposition on the eggshell was previously associated to reduce shell penetration by *Salmonella* and *Escherichia coli* (32), also serving as a water proofing agent and a barrier for invasion of bacterial and fungal pathogens (33). Cox et al. (34) reported that dirty eggs from broiler breeders may carry a high load of bacteria into the hatcher, which may result in low hatchability and high embryo mortality.

The improved cuticle deposition observed in the current experiment could be associated to the decreased contaminated eggs during the incubation when hens were fed the additive from QY biomass. In addition to the economic impact of contaminated eggs in the hatcheries, as reported by Messens et al. (35), eggshell contamination is also important for the consumers, where the lower shell microbial invasion level can lead to a lower occurrence of cross-contamination during food preparation. In the present study, the hatchability was not affected by the additives; however, it was observed lower contaminated and pipped eggs as well as lower early and middle mortality when hens were fed 250 g/ton QY blend. Alagawany et al. (36) evaluated a *Y. schidigera* extract for Japanese quails and observed that 200 g/ton improved egg production, hatchability of fertile eggs, and antioxidant and immunity functions. Indicating that these functions could contribute to the enhanced productive and reproductive performance.

In conclusion, hens fed a proprietary blend with polyphenols and saponins from *Yucca schidigera* and *Quillaja saponaria* achieved enhanced performance and egg quality compared to virginiamycin and non-supplemented feeds. Laying breeder hens fed diets supplemented with the Quillaja and Yucca additive maintained their productive performance. The Quillaja and Yucca supplementation also improved eggshell quality and cuticle deposition as well as reduced culled, dirty, and contaminated eggs in the hatchery.

## Data availability statement

The raw data supporting the conclusions of this article will be made available by the authors, without undue reservation.

## Ethics statement

The animal study was reviewed and approved by Ethics and Research Committee of the Federal University of Santa Maria.

## Author contributions

OS and CA: *in vivo* trial, data analysis, and trial conduction. JA and VB: *in vivo* trial and digestibility analysis. RM: yolk and shell strength analysis. LG and SB: draft and revision of the protocol and the paper. CS: draft and revision of the protocol, funding, trial conduction, draft and revision of the manuscript, and final approval for paper publication. All authors contributed to the article and approved the submitted version.
